# Supplementary material for: Experimental study on water-heat-salt migration and deformation characteristics of subgrade filler during freeze-thaw cycles in Northwestern China
Source: PLoS One. 2025 Dec 4;20(12):e0337173. doi: 10.1371/journal.pone.0337173 (PMC12677788; doi:10.1371/journal.pone.0337173)

Figure 2:

1.
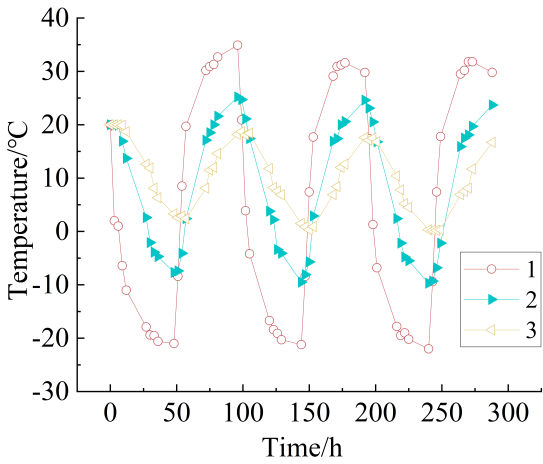


|  | 1 | 2 | 3 |
| --- | --- | --- | --- |
| 0 | 20 | 20 | 20 |
| 3 | 2 | 19.9 | 20 |
| 6 | 1 | 19.9 | 20 |
| 9 | -6.4 | 16.9 | 19.9 |
| 12 | -11 | 13.7 | 18.7 |
| 27 | -17.9 | 2.6 | 12.6 |
| 30 | -19.4 | -2.1 | 11.9 |
| 33 | -19.5 | -3.9 | 8.1 |
| 36 | -20.6 | -4.7 | 6.3 |
| 48 | -21 | -7.7 | 3.3 |
| 51 | -8.4 | -7.4 | 2.6 |
| 54 | 8.5 | -4.1 | 2.9 |
| 57 | 19.7 | 2.4 | 2.4 |
| 72 | 30.2 | 17.1 | 8.1 |
| 75 | 30.9 | 18.5 | 11.5 |
| 78 | 31.3 | 20 | 12 |
| 81 | 32.7 | 21.6 | 14.6 |
| 96 | 34.9 | 25.2 | 18.2 |
| 99 | 20.9 | 24.7 | 18.7 |
| 102 | 3.9 | 21.1 | 18.1 |
| 105 | -4.2 | 17.4 | 18.4 |
| 120 | -16.7 | 3.8 | 11.8 |
| 123 | -18.4 | 2.2 | 8.2 |
| 126 | -19.1 | -3.4 | 7.6 |
| 129 | -20.3 | -4.1 | 6.9 |
| 144 | -21.2 | -9.5 | 1.5 |
| 147 | -8.4 | -8.1 | 0.9 |
| 150 | 7.4 | -5.7 | 0.3 |
| 153 | 17.7 | 2.9 | 0.9 |
| 168 | 29.1 | 16.9 | 6.9 |
| 171 | 30.9 | 17.4 | 8.4 |
| 174 | 31.2 | 20 | 12 |
| 177 | 31.6 | 20.6 | 12.6 |
| 192 | 29.8 | 24.6 | 17.6 |
| 195 | 17.4 | 23.1 | 17.1 |
| 198 | 1.3 | 20.5 | 17.5 |
| 201 | -6.8 | 16.8 | 16.8 |
| 216 | -17.8 | 2.4 | 10.4 |
| 219 | -19.5 | -2.2 | 7.8 |
| 222 | -19 | -4.8 | 5.2 |
| 225 | -20.2 | -5.5 | 4.5 |
| 240 | -22 | -9.7 | 0.3 |
| 243 | -9.4 | -9.3 | 0.2 |
| 246 | 7.4 | -6.8 | 0.2 |
| 249 | 17.8 | -2.2 | 0.3 |
| 264 | 29.5 | 15.9 | 6.9 |
| 267 | 30.2 | 17.5 | 7.5 |
| 270 | 31.8 | 18.1 | 8.1 |
| 273 | 31.8 | 19.7 | 11.7 |
| 288 | 29.8 | 23.7 | 16.7 |
| 0 | 20 | 20 | 20 |

1.
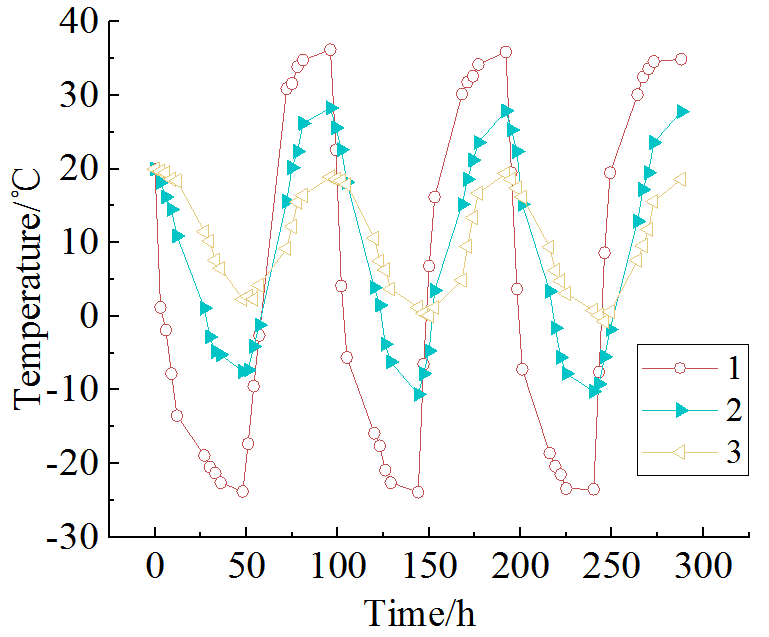


|  | 1 | 2 | 3 |
| --- | --- | --- | --- |
| 3 | 1.2 | 18.2 | 19.7 |
| 6 | -1.9 | 16.2 | 19.5 |
| 9 | -7.8 | 14.5 | 18.6 |
| 12 | -13.5 | 10.9 | 18.4 |
| 27 | -18.9 | 1.1 | 11.5 |
| 30 | -20.5 | -2.8 | 10.2 |
| 33 | -21.3 | -4.8 | 7.6 |
| 36 | -22.6 | -5.2 | 6.5 |
| 48 | -23.8 | -7.5 | 2.3 |
| 51 | -17.3 | -7.3 | 2.7 |
| 54 | -9.5 | -4.1 | 2.3 |
| 57 | -2.6 | -1.2 | 4.2 |
| 72 | 30.9 | 15.6 | 9.2 |
| 75 | 31.6 | 20.2 | 12.2 |
| 78 | 33.9 | 22.4 | 15.5 |
| 81 | 34.8 | 26.2 | 16.4 |
| 96 | 36.2 | 28.3 | 18.9 |
| 99 | 22.6 | 25.6 | 18.6 |
| 102 | 4.1 | 22.6 | 18.4 |
| 105 | -5.6 | 18.2 | 17.9 |
| 120 | -15.9 | 3.9 | 10.6 |
| 123 | -17.6 | 1.5 | 7.5 |
| 126 | -20.9 | -3.8 | 6.3 |
| 129 | -22.6 | -6.2 | 3.7 |
| 144 | -23.9 | -10.6 | 1.2 |
| 147 | -6.5 | -7.8 | 0.4 |
| 150 | 6.8 | -4.7 | 0.1 |
| 153 | 16.2 | 3.5 | 1.2 |
| 168 | 30.2 | 15.2 | 4.9 |
| 171 | 31.8 | 18.6 | 9.5 |
| 174 | 32.6 | 21.2 | 13.4 |
| 177 | 34.2 | 23.6 | 16.7 |
| 192 | 35.9 | 27.9 | 19.4 |
| 195 | 19.5 | 25.3 | 18.6 |
| 198 | 3.7 | 22.4 | 17.4 |
| 201 | -7.2 | 15.2 | 16.2 |
| 216 | -18.6 | 3.4 | 9.4 |
| 219 | -20.4 | -1.6 | 6.2 |
| 222 | -21.5 | -5.6 | 4.8 |
| 225 | -23.4 | -7.8 | 3.1 |
| 240 | -23.5 | -10.2 | 0.8 |
| 243 | -7.6 | -9.2 | 0.1 |
| 246 | 8.6 | -5.5 | -0.7 |
| 249 | 19.5 | -1.8 | 0.6 |
| 264 | 30.1 | 12.9 | 7.6 |
| 267 | 32.5 | 17.2 | 9.6 |
| 270 | 33.6 | 19.5 | 11.8 |
| 273 | 34.6 | 23.6 | 15.6 |
| 288 | 34.9 | 27.8 | 18.6 |
| 3 | 1.2 | 18.2 | 19.7 |

1.
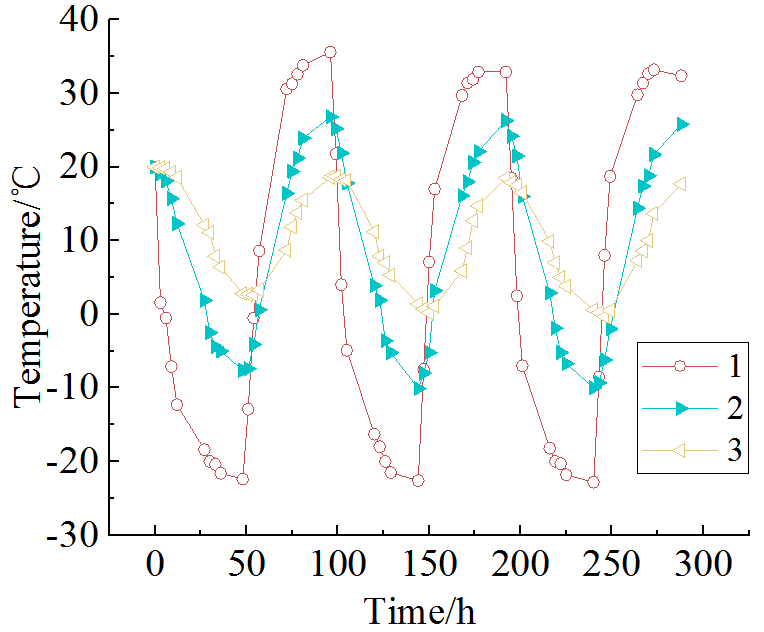


|  | 1 | 2 | 3 |
| --- | --- | --- | --- |
| 0 | 20 | 20 | 20 |
| 3 | 1.6 | 19.1 | 19.9 |
| 6 | -0.5 | 18.1 | 19.8 |
| 9 | -7.1 | 15.7 | 19.3 |
| 12 | -12.3 | 12.3 | 18.6 |
| 27 | -18.4 | 1.9 | 12.1 |
| 30 | -20 | -2.5 | 11.1 |
| 33 | -20.4 | -4.4 | 7.9 |
| 36 | -21.6 | -5 | 6.4 |
| 48 | -22.4 | -7.6 | 2.8 |
| 51 | -12.9 | -7.4 | 2.7 |
| 54 | -0.5 | -4.1 | 2.6 |
| 57 | 8.6 | 0.6 | 3.3 |
| 72 | 30.6 | 16.4 | 8.7 |
| 75 | 31.3 | 19.4 | 11.9 |
| 78 | 32.6 | 21.2 | 13.8 |
| 81 | 33.8 | 23.9 | 15.5 |
| 96 | 35.6 | 26.8 | 18.6 |
| 99 | 21.8 | 25.2 | 18.7 |
| 102 | 4 | 21.9 | 18.3 |
| 105 | -4.9 | 17.8 | 18.2 |
| 120 | -16.3 | 3.9 | 11.2 |
| 123 | -18 | 1.9 | 7.9 |
| 126 | -20 | -3.6 | 7 |
| 129 | -21.5 | -5.2 | 5.3 |
| 144 | -22.6 | -10.1 | 1.4 |
| 147 | -7.5 | -8 | 0.7 |
| 150 | 7.1 | -5.2 | 0.2 |
| 153 | 17 | 3.2 | 1.1 |
| 168 | 29.7 | 16.1 | 5.9 |
| 171 | 31.4 | 18 | 9 |
| 174 | 31.9 | 20.6 | 12.7 |
| 177 | 32.9 | 22.1 | 14.7 |
| 192 | 32.9 | 26.3 | 18.5 |
| 195 | 18.5 | 24.2 | 17.9 |
| 198 | 2.5 | 21.5 | 17.5 |
| 201 | -7 | 16 | 16.5 |
| 216 | -18.2 | 2.9 | 9.9 |
| 219 | -20 | -1.9 | 7 |
| 222 | -20.3 | -5.2 | 5 |
| 225 | -21.8 | -6.7 | 3.8 |
| 240 | -22.8 | -10 | 0.6 |
| 243 | -8.5 | -9.3 | 0.2 |
| 246 | 8 | -6.2 | -0.3 |
| 249 | 18.7 | -2 | 0.5 |
| 264 | 29.8 | 14.4 | 7.3 |
| 267 | 31.4 | 17.4 | 8.6 |
| 270 | 32.7 | 18.8 | 10 |
| 273 | 33.2 | 21.7 | 13.7 |
| 288 | 32.4 | 25.8 | 17.7 |

1.
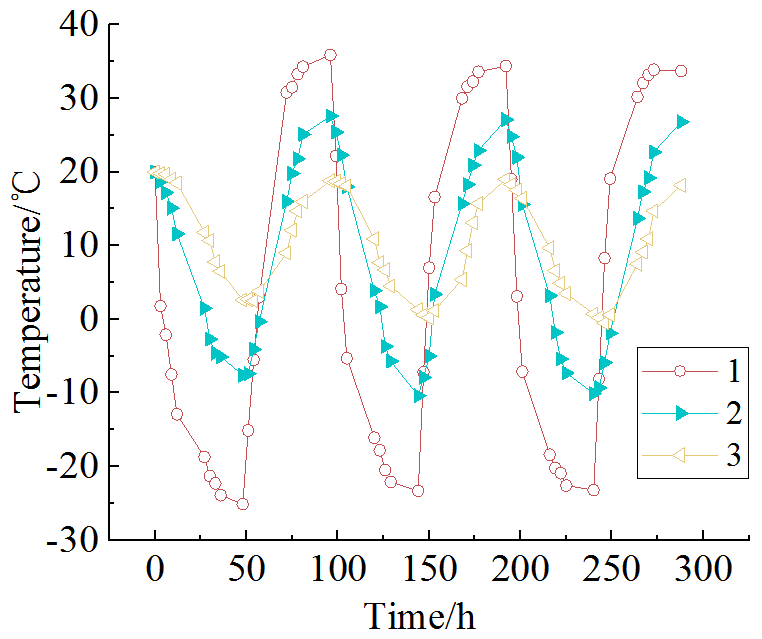


|  | 1 | 2 | 3 |
| --- | --- | --- | --- |
| 0 | 20 | 20 | 20 |
| 3 | 1.8 | 18.7 | 19.8 |
| 6 | -2.1 | 17.2 | 19.7 |
| 9 | -7.5 | 15.1 | 19 |
| 12 | -12.9 | 11.6 | 18.5 |
| 27 | -18.7 | 1.5 | 11.8 |
| 30 | -21.3 | -2.7 | 10.7 |
| 33 | -22.3 | -4.6 | 7.8 |
| 36 | -23.9 | -5.1 | 6.5 |
| 48 | -25.1 | -7.6 | 2.6 |
| 51 | -15.1 | -7.4 | 2.7 |
| 54 | -5.5 | -4.1 | 2.5 |
| 57 | 3 | -0.3 | 3.8 |
| 72 | 30.8 | 16 | 9 |
| 75 | 31.5 | 19.8 | 12.1 |
| 78 | 33.3 | 21.8 | 14.7 |
| 81 | 34.3 | 25.1 | 16 |
| 96 | 35.9 | 27.6 | 18.8 |
| 99 | 22.2 | 25.4 | 18.7 |
| 102 | 4.1 | 22.3 | 18.4 |
| 105 | -5.3 | 18 | 18.1 |
| 120 | -16.1 | 3.9 | 10.9 |
| 123 | -17.8 | 1.7 | 7.7 |
| 126 | -20.5 | -3.7 | 6.7 |
| 129 | -22.1 | -5.7 | 4.5 |
| 144 | -23.3 | -10.4 | 1.3 |
| 147 | -7.2 | -7.9 | 0.6 |
| 150 | 7 | -5 | 0.2 |
| 153 | 16.6 | 3.4 | 1.2 |
| 168 | 30 | 15.7 | 5.4 |
| 171 | 31.6 | 18.3 | 9.3 |
| 174 | 32.3 | 20.9 | 13.1 |
| 177 | 33.6 | 22.9 | 15.7 |
| 192 | 34.4 | 27.1 | 19 |
| 195 | 19 | 24.8 | 18.3 |
| 198 | 3.1 | 22 | 17.5 |
| 201 | -7.1 | 15.6 | 16.4 |
| 216 | -18.4 | 3.2 | 9.7 |
| 219 | -20.2 | -1.8 | 6.6 |
| 222 | -20.9 | -5.4 | 4.9 |
| 225 | -22.6 | -7.3 | 3.5 |
| 240 | -23.2 | -10.1 | 0.7 |
| 243 | -8.1 | -9.3 | 0.2 |
| 246 | 8.3 | -5.9 | -0.5 |
| 249 | 19.1 | -1.9 | 0.6 |
| 264 | 30.2 | 13.7 | 7.5 |
| 267 | 32.1 | 17.3 | 9.1 |
| 270 | 33.2 | 19.2 | 10.9 |
| 273 | 33.9 | 22.7 | 14.7 |
| 288 | 33.7 | 26.8 | 18.2 |

Figure3:

1.
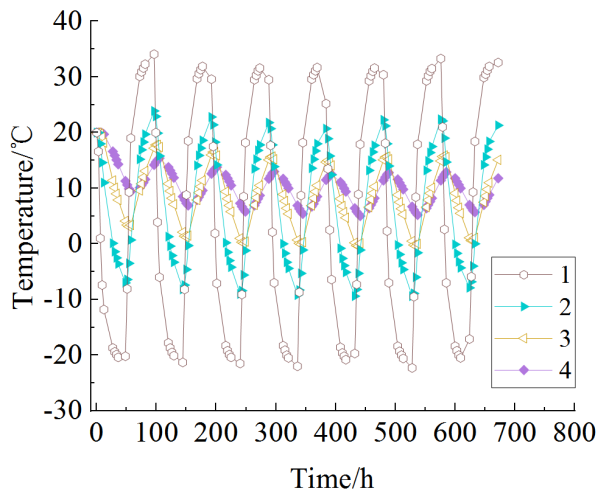


| Time | Temperature |  |  | Temperature | Temperature |  |  |  |
| --- | --- | --- | --- | --- | --- | --- | --- | --- |
| h | ℃ |  |  | ℃ | ℃ |  |  |  |
|  | 8 | 7 | 6 | 5 | 4 | 3 | 2 | 1 |
| 0 | 20 | 20 | 20 | 20 | 20 | 20 | 20 | 20 |
| 3 | 19.9 | 19.8 | 20 | 20 | 20 | 20 | 20 | 16.6 |
| 6 | 19.8 | 19.5 | 19.9 | 20 | 20 | 19.8 | 18 | 1 |
| 9 | 19.7 | 19.4 | 19.8 | 20 | 19.9 | 19.2 | 14.6 | -7.4 |
| 12 | 19.6 | 19.3 | 19.7 | 19.9 | 19.7 | 18.2 | 11 | -11.8 |
| 27 | 19.5 | 19.2 | 18.7 | 18.5 | 16.6 | 11.4 | 0.1 | -18.7 |
| 30 | 19.4 | 19 | 18.4 | 18.1 | 15.9 | 10.1 | -1.4 | -19.4 |
| 33 | 19.3 | 18.8 | 18.1 | 17.6 | 15.1 | 9 | -2.5 | -19.9 |
| 36 | 19.2 | 18.6 | 17.8 | 17.1 | 14.3 | 7.9 | -3.6 | -20.4 |
| 48 | 19.2 | 18 | 16.6 | 15.1 | 11.3 | 4.1 | -7 | -20.2 |
| 51 | 19.2 | 17.8 | 16.2 | 14.6 | 10.6 | 3.4 | -6.4 | -8.1 |
| 54 | 19.1 | 17.6 | 15.9 | 14.1 | 9.9 | 3.1 | -3.5 | 9.3 |
| 57 | 18.9 | 17.4 | 15.5 | 13.6 | 9.4 | 3.4 | 0.7 | 19 |
| 72 | 18.7 | 16.9 | 14.2 | 12.3 | 10.1 | 9.6 | 15.2 | 30.1 |
| 75 | 18.6 | 16.5 | 14 | 12.3 | 10.5 | 10.8 | 16.9 | 30.9 |
| 78 | 18.5 | 16.4 | 13.9 | 12.4 | 11 | 12 | 18.3 | 31.6 |
| 81 | 18.4 | 16.3 | 13.8 | 12.5 | 11.6 | 13.1 | 19.7 | 32.3 |
| 96 | 18.3 | 16 | 13.9 | 13.5 | 14.2 | 17.2 | 23.9 | 34.1 |
| 99 | 18.3 | 15.8 | 13.9 | 13.7 | 14.7 | 17.8 | 22.9 | 20 |
| 102 | 18.3 | 15.9 | 14 | 14 | 15.1 | 17.8 | 19.9 | 3.9 |
| 105 | 18.4 | 16 | 14.1 | 14.2 | 15.4 | 17.4 | 15.8 | -6 |
| 120 | 18.4 | 16.1 | 15 | 14.3 | 13.8 | 10.8 | 1.3 | -17.8 |
| 123 | 18.5 | 16.2 | 15.5 | 14.2 | 13.2 | 9.5 | -0.4 | -18.7 |
| 126 | 18.5 | 16.3 | 15.6 | 13.9 | 12.6 | 8.2 | -2.1 | -19.5 |
| 129 | 18.6 | 16.4 | 15.4 | 13.6 | 11.9 | 7.1 | -3.3 | -20.1 |
| 144 | 18.6 | 16.4 | 15 | 11.9 | 8.5 | 2.1 | -8.2 | -21.3 |
| 147 | 18.6 | 16.3 | 14.9 | 11.5 | 7.9 | 1.4 | -7.4 | -8.2 |
| 150 | 18.6 | 16.2 | 14.8 | 11.1 | 7.3 | 1.2 | -4.6 | 8.8 |
| 153 | 18.5 | 15.9 | 14.7 | 10.7 | 6.9 | 1.5 | -0.3 | 18.5 |
| 168 | 18.5 | 15.6 | 13 | 10 | 7.9 | 7.9 | 14.1 | 29.7 |
| 171 | 18.4 | 15.4 | 12.8 | 10 | 8.4 | 9.2 | 15.9 | 30.6 |
| 174 | 18.4 | 15.1 | 12.6 | 10.2 | 9 | 10.3 | 17.2 | 31.3 |
| 177 | 18.3 | 14.8 | 12.5 | 10.4 | 9.6 | 11.4 | 18.6 | 31.9 |
| 192 | 18.2 | 14.7 | 12.8 | 11.7 | 12.6 | 16 | 22.8 | 29.6 |
| 195 | 18.3 | 14.8 | 13 | 12 | 13.1 | 16.5 | 21.4 | 17.5 |
| 198 | 18.2 | 14.9 | 13.4 | 12.3 | 13.5 | 16.5 | 18.3 | 1.9 |
| 201 | 18.3 | 15 | 13.6 | 12.6 | 13.8 | 15.9 | 14.2 | -7.1 |
| 216 | 18.4 | 15.2 | 14.2 | 12.9 | 12.4 | 9.4 | 0.2 | -18.3 |
| 219 | 18.4 | 15.4 | 14.2 | 12.8 | 11.8 | 8.1 | -1.5 | -19.2 |
| 222 | 18.5 | 15.6 | 14.3 | 12.6 | 11.2 | 6.9 | -3 | -19.9 |
| 225 | 18.5 | 15.8 | 14.3 | 12.3 | 10.5 | 5.8 | -4.2 | -20.4 |
| 240 | 18.5 | 15.8 | 14.4 | 10.8 | 7.3 | 1 | -9 | -21.5 |
| 243 | 18.6 | 15.6 | 14.2 | 10.4 | 6.7 | 0.3 | -8.4 | -9.1 |
| 246 | 18.5 | 15.4 | 13.5 | 10.1 | 6.2 | 0.1 | -5.6 | 8.5 |
| 249 | 18.5 | 15.2 | 13.2 | 9.7 | 5.8 | 0.4 | -1.2 | 18.2 |
| 264 | 18.4 | 14.8 | 12.8 | 9.1 | 6.9 | 7 | 13.5 | 29.5 |
| 267 | 18.3 | 14.7 | 12.2 | 9.2 | 7.5 | 8.3 | 15.2 | 30.3 |
| 270 | 18.3 | 14.7 | 12 | 9.3 | 8.1 | 9.6 | 16.8 | 31.1 |
| 273 | 18.2 | 14.6 | 11.8 | 9.6 | 8.7 | 10.5 | 17.8 | 31.6 |
| 288 | 18.1 | 14.5 | 11.7 | 11 | 11.7 | 15.1 | 21.8 | 29.5 |
| 291 | 18 | 14.4 | 11.7 | 11.3 | 12.2 | 15.6 | 20.7 | 17.7 |
| 294 | 17.9 | 14.6 | 12.2 | 11.6 | 12.7 | 15.7 | 17.8 | 2.1 |
| 297 | 17.8 | 14.8 | 12.6 | 11.9 | 13 | 15.2 | 13.9 | -7 |
| 312 | 18 | 15.4 | 13.5 | 12.3 | 11.7 | 8.9 | 0 | -18.3 |
| 315 | 18.1 | 15.6 | 14.2 | 12.2 | 11.1 | 7.7 | -1.7 | -19.1 |
| 318 | 18.2 | 15.8 | 14.1 | 12 | 10.6 | 6.4 | -3.3 | -20 |
| 321 | 18.3 | 16 | 14 | 11.8 | 10 | 5.4 | -4.4 | -20.5 |
| 336 | 18.4 | 16 | 14 | 10.3 | 6.9 | 0.7 | -9.2 | -22 |
| 339 | 18.5 | 15.8 | 14.1 | 10 | 6.3 | 0.1 | -8.3 | -8.7 |
| 342 | 18.4 | 15.7 | 13.7 | 9.6 | 5.8 | -0.1 | -5.3 | 8.7 |
| 345 | 18.3 | 15.6 | 13.4 | 9.3 | 5.4 | 0.3 | -1.1 | 18.2 |
| 360 | 18.3 | 15.2 | 12.2 | 8.7 | 6.7 | 6.9 | 13.6 | 29.6 |
| 363 | 18.2 | 15.1 | 12 | 8.9 | 7.2 | 8.2 | 15.2 | 30.4 |
| 366 | 18.1 | 15 | 12 | 9 | 7.9 | 9.4 | 16.7 | 31.1 |
| 369 | 17.8 | 14.9 | 12.4 | 9.3 | 8.5 | 10.5 | 18 | 31.7 |
| 384 | 17.8 | 14.8 | 13.1 | 10.8 | 11.5 | 14.7 | 20.7 | 25.2 |
| 387 | 17.8 | 14.7 | 13.3 | 11.1 | 11.9 | 15 | 18.9 | 13.5 |
| 390 | 17.8 | 14.6 | 13.6 | 11.4 | 12.3 | 14.7 | 15.8 | 2.5 |
| 393 | 17.9 | 14.7 | 13.9 | 11.6 | 12.5 | 14.1 | 12.4 | -6.4 |
| 408 | 18.1 | 14.9 | 14.5 | 11.9 | 11.1 | 8.1 | -0.8 | -18.6 |
| 411 | 18.2 | 15.2 | 14.9 | 11.8 | 10.6 | 6.8 | -2.6 | -19.6 |
| 414 | 18.3 | 15.4 | 15.2 | 11.6 | 10 | 5.7 | -3.9 | -20.2 |
| 417 | 18.3 | 15.7 | 15.1 | 11.4 | 9.4 | 4.7 | -5.1 | -20.8 |
| 432 | 18.4 | 15.9 | 14.5 | 9.9 | 6.4 | 0.2 | -9.4 | -19.7 |
| 435 | 18.4 | 16.1 | 14 | 9.6 | 5.8 | -0.3 | -8.2 | -7.3 |
| 438 | 18.3 | 15.9 | 13.8 | 9.3 | 5.4 | -0.4 | -5.3 | 8.9 |
| 441 | 18.3 | 15.7 | 13.6 | 8.9 | 5 | 0.1 | -1.1 | 17.9 |
| 456 | 18.2 | 15.2 | 12.4 | 8.5 | 6.4 | 6.6 | 13.2 | 29.3 |
| 459 | 18.1 | 15.2 | 12.3 | 8.6 | 7 | 8 | 15 | 30.2 |
| 462 | 18.1 | 15.1 | 12.2 | 8.8 | 7.6 | 9.1 | 16.5 | 31 |
| 465 | 17.9 | 15 | 12.1 | 9.1 | 8.2 | 10.3 | 17.7 | 31.6 |
| 480 | 17.9 | 14.9 | 11.9 | 10.5 | 11.4 | 15.1 | 22.3 | 30.4 |
| 483 | 18 | 14.8 | 11.8 | 10.9 | 12 | 15.6 | 21.2 | 18.1 |
| 486 | 18.1 | 14.7 | 11.9 | 11.2 | 12.5 | 15.7 | 18 | 2.3 |
| 489 | 18.1 | 14.8 | 12.2 | 11.6 | 12.8 | 15.2 | 14 | -7 |
| 504 | 18.1 | 14.9 | 13 | 12.1 | 11.6 | 8.8 | -0.2 | -18.4 |
| 507 | 18.1 | 15.2 | 13.2 | 12 | 11 | 7.5 | -1.9 | -19.3 |
| 510 | 18.2 | 15.5 | 13.6 | 11.8 | 10.4 | 6.3 | -3.4 | -20 |
| 513 | 18.2 | 15.6 | 13.8 | 11.6 | 9.8 | 5.3 | -4.6 | -20.6 |
| 528 | 18.3 | 15.8 | 13.6 | 10.2 | 6.7 | 0.5 | -9.4 | -22.3 |
| 531 | 18.3 | 15.7 | 13.4 | 9.8 | 6.1 | -0.2 | -8.9 | -9.5 |
| 534 | 18.4 | 15.7 | 13.3 | 9.5 | 5.6 | -0.4 | -6 | 8.4 |
| 537 | 18.2 | 15.6 | 13.2 | 9.1 | 5.2 | 0 | -1.6 | 17.9 |
| 552 | 18.2 | 15.2 | 12.5 | 8.6 | 6.4 | 6.6 | 13.2 | 29.3 |
| 555 | 18.1 | 15.2 | 12.4 | 8.7 | 7 | 7.9 | 14.9 | 30.2 |
| 558 | 18 | 15.1 | 12.3 | 8.9 | 7.6 | 9.1 | 16.4 | 31 |
| 561 | 18 | 14.9 | 12.2 | 9.1 | 8.2 | 10.1 | 17.5 | 31.5 |
| 576 | 17.9 | 14.8 | 12.2 | 10.6 | 11.4 | 14.9 | 22.4 | 33.3 |
| 579 | 17.9 | 14.8 | 12.4 | 10.9 | 12 | 15.7 | 22.1 | 21 |
| 582 | 17.9 | 14.7 | 12.6 | 11.3 | 12.5 | 15.9 | 19 | 2.6 |
| 585 | 17.9 | 14.8 | 12.8 | 11.6 | 12.9 | 15.6 | 14.7 | -7 |
| 600 | 18 | 14.9 | 13.9 | 12.3 | 11.8 | 9.1 | -0.1 | -18.4 |
| 603 | 18.1 | 15 | 13.9 | 12.1 | 11.2 | 7.8 | -1.7 | -19.2 |
| 606 | 18.2 | 15.2 | 14 | 12 | 10.6 | 6.5 | -3.2 | -20 |
| 609 | 18.2 | 15.4 | 14.1 | 11.7 | 10 | 5.5 | -4.3 | -20.5 |
| 624 | 18.2 | 15.6 | 14.2 | 10.3 | 7 | 1.1 | -7.9 | -17.1 |
| 627 | 18.3 | 15.7 | 14.1 | 10 | 6.5 | 0.6 | -6.8 | -5.9 |
| 630 | 18.3 | 15.6 | 13.8 | 9.6 | 6 | 0.6 | -4 | 9.3 |
| 633 | 18.3 | 15.4 | 13.5 | 9.3 | 5.7 | 1.1 | 0 | 18.4 |
| 648 | 18.3 | 15.2 | 12.5 | 8.9 | 7 | 7.5 | 14.2 | 29.9 |
| 651 | 18.3 | 15.1 | 12.1 | 9 | 7.6 | 8.6 | 15.6 | 30.5 |
| 654 | 18.3 | 15.1 | 12.4 | 9.2 | 8.2 | 9.8 | 17 | 31.2 |
| 657 | 18.3 | 15.1 | 12.5 | 9.4 | 8.8 | 11 | 18.4 | 31.9 |
| 672 | 18.2 | 15 | 12.6 | 10.9 | 11.8 | 15.1 | 21.3 | 32.6 |

1.
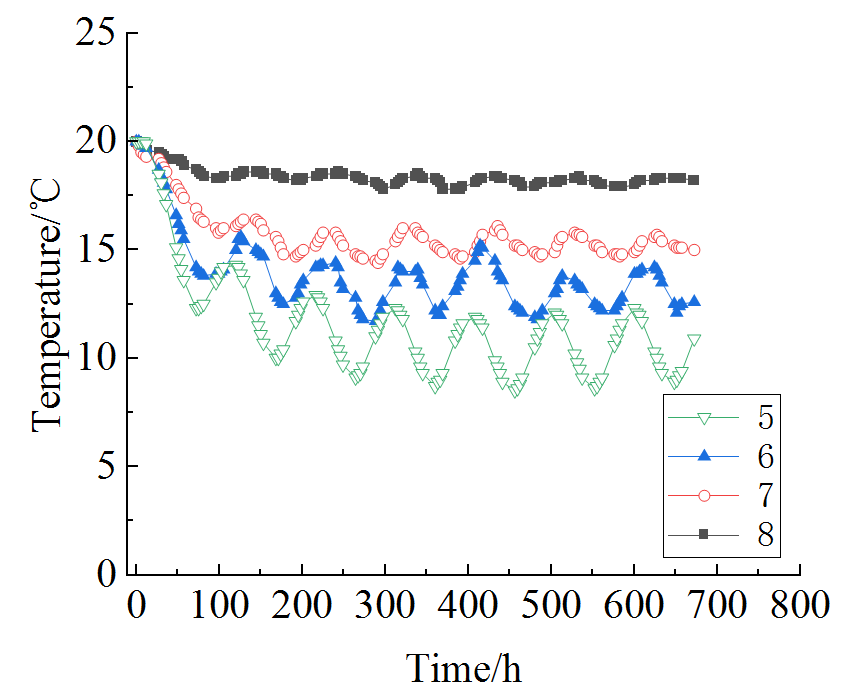


| Time | Temperature |  |  | Temperature | Temperature |  |  |  |
| --- | --- | --- | --- | --- | --- | --- | --- | --- |
| h | ℃ |  |  | ℃ | ℃ |  |  |  |
|  | 8 | 7 | 6 | 5 | 4 | 3 | 2 | 1 |
| 0 | 20 | 20 | 20 | 20 | 20 | 20 | 20 | 20 |
| 3 | 19.9 | 19.8 | 20 | 20 | 20 | 20 | 20 | 16.6 |
| 6 | 19.8 | 19.5 | 19.9 | 20 | 20 | 19.8 | 18 | 1 |
| 9 | 19.7 | 19.4 | 19.8 | 20 | 19.9 | 19.2 | 14.6 | -7.4 |
| 12 | 19.6 | 19.3 | 19.7 | 19.9 | 19.7 | 18.2 | 11 | -11.8 |
| 27 | 19.5 | 19.2 | 18.7 | 18.5 | 16.6 | 11.4 | 0.1 | -18.7 |
| 30 | 19.4 | 19 | 18.4 | 18.1 | 15.9 | 10.1 | -1.4 | -19.4 |
| 33 | 19.3 | 18.8 | 18.1 | 17.6 | 15.1 | 9 | -2.5 | -19.9 |
| 36 | 19.2 | 18.6 | 17.8 | 17.1 | 14.3 | 7.9 | -3.6 | -20.4 |
| 48 | 19.2 | 18 | 16.6 | 15.1 | 11.3 | 4.1 | -7 | -20.2 |
| 51 | 19.2 | 17.8 | 16.2 | 14.6 | 10.6 | 3.4 | -6.4 | -8.1 |
| 54 | 19.1 | 17.6 | 15.9 | 14.1 | 9.9 | 3.1 | -3.5 | 9.3 |
| 57 | 18.9 | 17.4 | 15.5 | 13.6 | 9.4 | 3.4 | 0.7 | 19 |
| 72 | 18.7 | 16.9 | 14.2 | 12.3 | 10.1 | 9.6 | 15.2 | 30.1 |
| 75 | 18.6 | 16.5 | 14 | 12.3 | 10.5 | 10.8 | 16.9 | 30.9 |
| 78 | 18.5 | 16.4 | 13.9 | 12.4 | 11 | 12 | 18.3 | 31.6 |
| 81 | 18.4 | 16.3 | 13.8 | 12.5 | 11.6 | 13.1 | 19.7 | 32.3 |
| 96 | 18.3 | 16 | 13.9 | 13.5 | 14.2 | 17.2 | 23.9 | 34.1 |
| 99 | 18.3 | 15.8 | 13.9 | 13.7 | 14.7 | 17.8 | 22.9 | 20 |
| 102 | 18.3 | 15.9 | 14 | 14 | 15.1 | 17.8 | 19.9 | 3.9 |
| 105 | 18.4 | 16 | 14.1 | 14.2 | 15.4 | 17.4 | 15.8 | -6 |
| 120 | 18.4 | 16.1 | 15 | 14.3 | 13.8 | 10.8 | 1.3 | -17.8 |
| 123 | 18.5 | 16.2 | 15.5 | 14.2 | 13.2 | 9.5 | -0.4 | -18.7 |
| 126 | 18.5 | 16.3 | 15.6 | 13.9 | 12.6 | 8.2 | -2.1 | -19.5 |
| 129 | 18.6 | 16.4 | 15.4 | 13.6 | 11.9 | 7.1 | -3.3 | -20.1 |
| 144 | 18.6 | 16.4 | 15 | 11.9 | 8.5 | 2.1 | -8.2 | -21.3 |
| 147 | 18.6 | 16.3 | 14.9 | 11.5 | 7.9 | 1.4 | -7.4 | -8.2 |
| 150 | 18.6 | 16.2 | 14.8 | 11.1 | 7.3 | 1.2 | -4.6 | 8.8 |
| 153 | 18.5 | 15.9 | 14.7 | 10.7 | 6.9 | 1.5 | -0.3 | 18.5 |
| 168 | 18.5 | 15.6 | 13 | 10 | 7.9 | 7.9 | 14.1 | 29.7 |
| 171 | 18.4 | 15.4 | 12.8 | 10 | 8.4 | 9.2 | 15.9 | 30.6 |
| 174 | 18.4 | 15.1 | 12.6 | 10.2 | 9 | 10.3 | 17.2 | 31.3 |
| 177 | 18.3 | 14.8 | 12.5 | 10.4 | 9.6 | 11.4 | 18.6 | 31.9 |
| 192 | 18.2 | 14.7 | 12.8 | 11.7 | 12.6 | 16 | 22.8 | 29.6 |
| 195 | 18.3 | 14.8 | 13 | 12 | 13.1 | 16.5 | 21.4 | 17.5 |
| 198 | 18.2 | 14.9 | 13.4 | 12.3 | 13.5 | 16.5 | 18.3 | 1.9 |
| 201 | 18.3 | 15 | 13.6 | 12.6 | 13.8 | 15.9 | 14.2 | -7.1 |
| 216 | 18.4 | 15.2 | 14.2 | 12.9 | 12.4 | 9.4 | 0.2 | -18.3 |
| 219 | 18.4 | 15.4 | 14.2 | 12.8 | 11.8 | 8.1 | -1.5 | -19.2 |
| 222 | 18.5 | 15.6 | 14.3 | 12.6 | 11.2 | 6.9 | -3 | -19.9 |
| 225 | 18.5 | 15.8 | 14.3 | 12.3 | 10.5 | 5.8 | -4.2 | -20.4 |
| 240 | 18.5 | 15.8 | 14.4 | 10.8 | 7.3 | 1 | -9 | -21.5 |
| 243 | 18.6 | 15.6 | 14.2 | 10.4 | 6.7 | 0.3 | -8.4 | -9.1 |
| 246 | 18.5 | 15.4 | 13.5 | 10.1 | 6.2 | 0.1 | -5.6 | 8.5 |
| 249 | 18.5 | 15.2 | 13.2 | 9.7 | 5.8 | 0.4 | -1.2 | 18.2 |
| 264 | 18.4 | 14.8 | 12.8 | 9.1 | 6.9 | 7 | 13.5 | 29.5 |
| 267 | 18.3 | 14.7 | 12.2 | 9.2 | 7.5 | 8.3 | 15.2 | 30.3 |
| 270 | 18.3 | 14.7 | 12 | 9.3 | 8.1 | 9.6 | 16.8 | 31.1 |
| 273 | 18.2 | 14.6 | 11.8 | 9.6 | 8.7 | 10.5 | 17.8 | 31.6 |
| 288 | 18.1 | 14.5 | 11.7 | 11 | 11.7 | 15.1 | 21.8 | 29.5 |
| 291 | 18 | 14.4 | 11.7 | 11.3 | 12.2 | 15.6 | 20.7 | 17.7 |
| 294 | 17.9 | 14.6 | 12.2 | 11.6 | 12.7 | 15.7 | 17.8 | 2.1 |
| 297 | 17.8 | 14.8 | 12.6 | 11.9 | 13 | 15.2 | 13.9 | -7 |
| 312 | 18 | 15.4 | 13.5 | 12.3 | 11.7 | 8.9 | 0 | -18.3 |
| 315 | 18.1 | 15.6 | 14.2 | 12.2 | 11.1 | 7.7 | -1.7 | -19.1 |
| 318 | 18.2 | 15.8 | 14.1 | 12 | 10.6 | 6.4 | -3.3 | -20 |
| 321 | 18.3 | 16 | 14 | 11.8 | 10 | 5.4 | -4.4 | -20.5 |
| 336 | 18.4 | 16 | 14 | 10.3 | 6.9 | 0.7 | -9.2 | -22 |
| 339 | 18.5 | 15.8 | 14.1 | 10 | 6.3 | 0.1 | -8.3 | -8.7 |
| 342 | 18.4 | 15.7 | 13.7 | 9.6 | 5.8 | -0.1 | -5.3 | 8.7 |
| 345 | 18.3 | 15.6 | 13.4 | 9.3 | 5.4 | 0.3 | -1.1 | 18.2 |
| 360 | 18.3 | 15.2 | 12.2 | 8.7 | 6.7 | 6.9 | 13.6 | 29.6 |
| 363 | 18.2 | 15.1 | 12 | 8.9 | 7.2 | 8.2 | 15.2 | 30.4 |
| 366 | 18.1 | 15 | 12 | 9 | 7.9 | 9.4 | 16.7 | 31.1 |
| 369 | 17.8 | 14.9 | 12.4 | 9.3 | 8.5 | 10.5 | 18 | 31.7 |
| 384 | 17.8 | 14.8 | 13.1 | 10.8 | 11.5 | 14.7 | 20.7 | 25.2 |
| 387 | 17.8 | 14.7 | 13.3 | 11.1 | 11.9 | 15 | 18.9 | 13.5 |
| 390 | 17.8 | 14.6 | 13.6 | 11.4 | 12.3 | 14.7 | 15.8 | 2.5 |
| 393 | 17.9 | 14.7 | 13.9 | 11.6 | 12.5 | 14.1 | 12.4 | -6.4 |
| 408 | 18.1 | 14.9 | 14.5 | 11.9 | 11.1 | 8.1 | -0.8 | -18.6 |
| 411 | 18.2 | 15.2 | 14.9 | 11.8 | 10.6 | 6.8 | -2.6 | -19.6 |
| 414 | 18.3 | 15.4 | 15.2 | 11.6 | 10 | 5.7 | -3.9 | -20.2 |
| 417 | 18.3 | 15.7 | 15.1 | 11.4 | 9.4 | 4.7 | -5.1 | -20.8 |
| 432 | 18.4 | 15.9 | 14.5 | 9.9 | 6.4 | 0.2 | -9.4 | -19.7 |
| 435 | 18.4 | 16.1 | 14 | 9.6 | 5.8 | -0.3 | -8.2 | -7.3 |
| 438 | 18.3 | 15.9 | 13.8 | 9.3 | 5.4 | -0.4 | -5.3 | 8.9 |
| 441 | 18.3 | 15.7 | 13.6 | 8.9 | 5 | 0.1 | -1.1 | 17.9 |
| 456 | 18.2 | 15.2 | 12.4 | 8.5 | 6.4 | 6.6 | 13.2 | 29.3 |
| 459 | 18.1 | 15.2 | 12.3 | 8.6 | 7 | 8 | 15 | 30.2 |
| 462 | 18.1 | 15.1 | 12.2 | 8.8 | 7.6 | 9.1 | 16.5 | 31 |
| 465 | 17.9 | 15 | 12.1 | 9.1 | 8.2 | 10.3 | 17.7 | 31.6 |
| 480 | 17.9 | 14.9 | 11.9 | 10.5 | 11.4 | 15.1 | 22.3 | 30.4 |
| 483 | 18 | 14.8 | 11.8 | 10.9 | 12 | 15.6 | 21.2 | 18.1 |
| 486 | 18.1 | 14.7 | 11.9 | 11.2 | 12.5 | 15.7 | 18 | 2.3 |
| 489 | 18.1 | 14.8 | 12.2 | 11.6 | 12.8 | 15.2 | 14 | -7 |
| 504 | 18.1 | 14.9 | 13 | 12.1 | 11.6 | 8.8 | -0.2 | -18.4 |
| 507 | 18.1 | 15.2 | 13.2 | 12 | 11 | 7.5 | -1.9 | -19.3 |
| 510 | 18.2 | 15.5 | 13.6 | 11.8 | 10.4 | 6.3 | -3.4 | -20 |
| 513 | 18.2 | 15.6 | 13.8 | 11.6 | 9.8 | 5.3 | -4.6 | -20.6 |
| 528 | 18.3 | 15.8 | 13.6 | 10.2 | 6.7 | 0.5 | -9.4 | -22.3 |
| 531 | 18.3 | 15.7 | 13.4 | 9.8 | 6.1 | -0.2 | -8.9 | -9.5 |
| 534 | 18.4 | 15.7 | 13.3 | 9.5 | 5.6 | -0.4 | -6 | 8.4 |
| 537 | 18.2 | 15.6 | 13.2 | 9.1 | 5.2 | 0 | -1.6 | 17.9 |
| 552 | 18.2 | 15.2 | 12.5 | 8.6 | 6.4 | 6.6 | 13.2 | 29.3 |
| 555 | 18.1 | 15.2 | 12.4 | 8.7 | 7 | 7.9 | 14.9 | 30.2 |
| 558 | 18 | 15.1 | 12.3 | 8.9 | 7.6 | 9.1 | 16.4 | 31 |
| 561 | 18 | 14.9 | 12.2 | 9.1 | 8.2 | 10.1 | 17.5 | 31.5 |
| 576 | 17.9 | 14.8 | 12.2 | 10.6 | 11.4 | 14.9 | 22.4 | 33.3 |
| 579 | 17.9 | 14.8 | 12.4 | 10.9 | 12 | 15.7 | 22.1 | 21 |
| 582 | 17.9 | 14.7 | 12.6 | 11.3 | 12.5 | 15.9 | 19 | 2.6 |
| 585 | 17.9 | 14.8 | 12.8 | 11.6 | 12.9 | 15.6 | 14.7 | -7 |
| 600 | 18 | 14.9 | 13.9 | 12.3 | 11.8 | 9.1 | -0.1 | -18.4 |
| 603 | 18.1 | 15 | 13.9 | 12.1 | 11.2 | 7.8 | -1.7 | -19.2 |
| 606 | 18.2 | 15.2 | 14 | 12 | 10.6 | 6.5 | -3.2 | -20 |
| 609 | 18.2 | 15.4 | 14.1 | 11.7 | 10 | 5.5 | -4.3 | -20.5 |
| 624 | 18.2 | 15.6 | 14.2 | 10.3 | 7 | 1.1 | -7.9 | -17.1 |
| 627 | 18.3 | 15.7 | 14.1 | 10 | 6.5 | 0.6 | -6.8 | -5.9 |
| 630 | 18.3 | 15.6 | 13.8 | 9.6 | 6 | 0.6 | -4 | 9.3 |
| 633 | 18.3 | 15.4 | 13.5 | 9.3 | 5.7 | 1.1 | 0 | 18.4 |
| 648 | 18.3 | 15.2 | 12.5 | 8.9 | 7 | 7.5 | 14.2 | 29.9 |
| 651 | 18.3 | 15.1 | 12.1 | 9 | 7.6 | 8.6 | 15.6 | 30.5 |
| 654 | 18.3 | 15.1 | 12.4 | 9.2 | 8.2 | 9.8 | 17 | 31.2 |
| 657 | 18.3 | 15.1 | 12.5 | 9.4 | 8.8 | 11 | 18.4 | 31.9 |
| 672 | 18.2 | 15 | 12.6 | 10.9 | 11.8 | 15.1 | 21.3 | 32.6 |

Figure4:


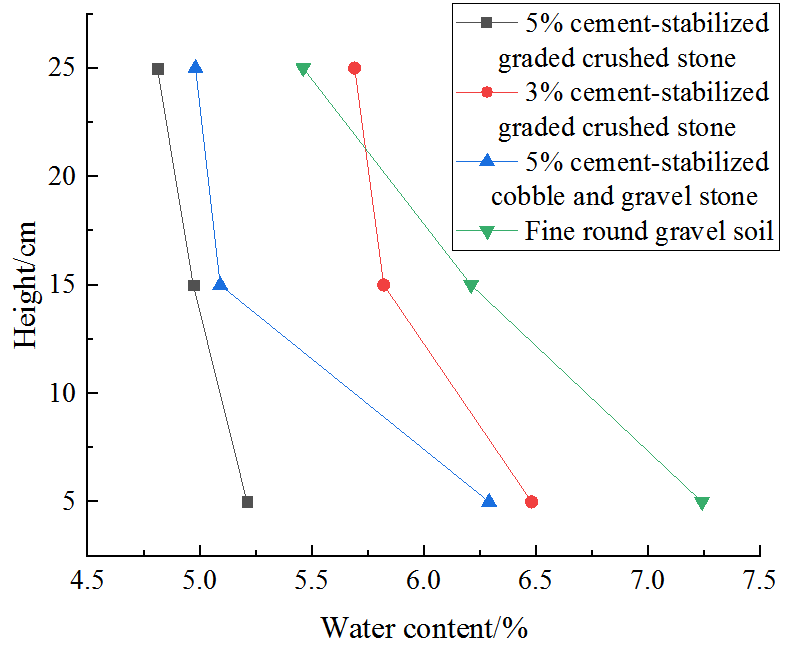


| Height | Water content |  |  |  |
| --- | --- | --- | --- | --- |
| cm | % |  |  |  |
|  | 5% cement-stabilized graded crushed stone | 3% cement-stabilized graded crushed stone | 5% cement-stabilized cobble and gravel stone | Fine round gravel stone |
| 25 | 4.81 | 5.69 | 4.98 | 5.46 |
| 15 | 4.97 | 5.82 | 5.09 | 6.21 |
| 5 | 5.21 | 6.48 | 6.29 | 7.24 |

Figure 5:


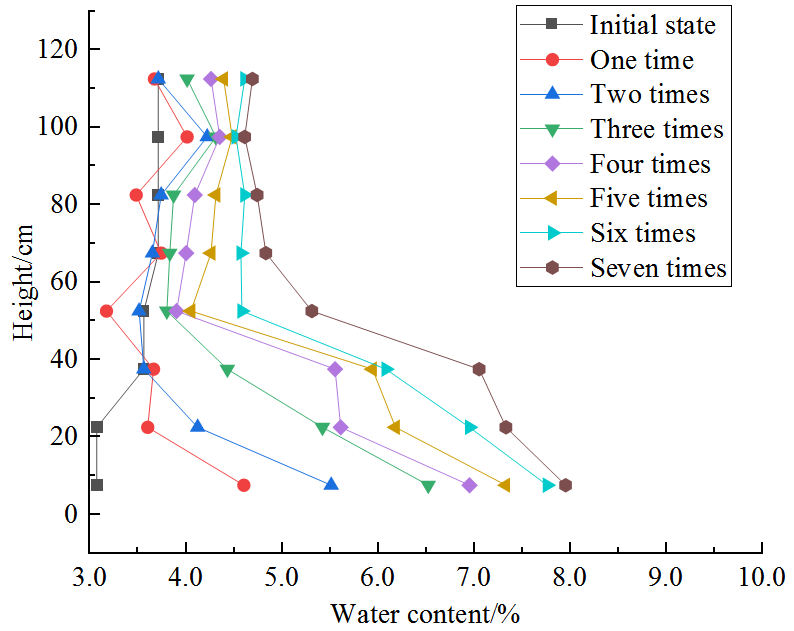


| Height | Water content |  |  |  |  |  |  |  |
| --- | --- | --- | --- | --- | --- | --- | --- | --- |
| cm | % |  |  |  |  |  |  |  |
|  | Initial time | One time | Two times | Three times | Four times | Five times | Six  times | Seven times |
| 112.5 | 3.71 | 3.67 | 3.71 | 4.01 | 4.26 | 4.39 | 4.61 | 4.69 |
| 97.5 | 3.71 | 4.01 | 4.22 | 4.31 | 4.35 | 4.48 | 4.52 | 4.61 |
| 82.5 | 3.71 | 3.48 | 3.74 | 3.87 | 4.09 | 4.31 | 4.61 | 4.74 |
| 67.5 | 3.71 | 3.74 | 3.65 | 3.83 | 4 | 4.26 | 4.57 | 4.83 |
| 52.5 | 3.56 | 3.17 | 3.51 | 3.8 | 3.9 | 4.05 | 4.58 | 5.31 |
| 37.5 | 3.56 | 3.66 | 3.56 | 4.43 | 5.55 | 5.94 | 6.08 | 7.05 |
| 22.5 | 3.07 | 3.6 | 4.12 | 5.42 | 5.61 | 6.18 | 6.95 | 7.33 |
| 7.5 | 3.07 | 4.6 | 5.51 | 6.52 | 6.95 | 7.33 | 7.76 | 7.95 |

Figure 8:


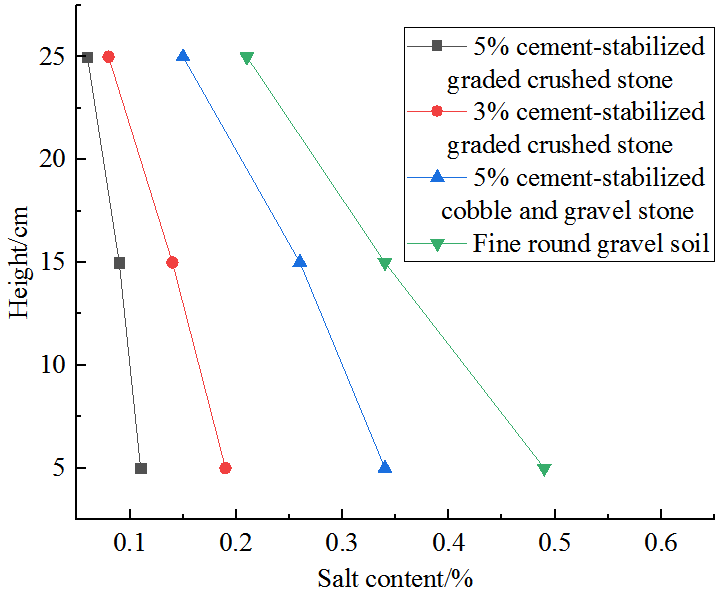


| Height | Water content |  |  |  |
| --- | --- | --- | --- | --- |
| cm | % |  |  |  |
|  | 5% cement-stabilized graded crushed stone | 3% cement-stabilized graded crushed stone | 5% cement-stabilized cobble and gravel stone | Fine round gravel stone |
| 25 | 0.06 | 0.08 | 0.15 | 0.21 |
| 15 | 0.09 | 0.14 | 0.26 | 0.34 |
| 5 | 0.11 | 0.19 | 0.34 | 0.49 |

Figure 9:


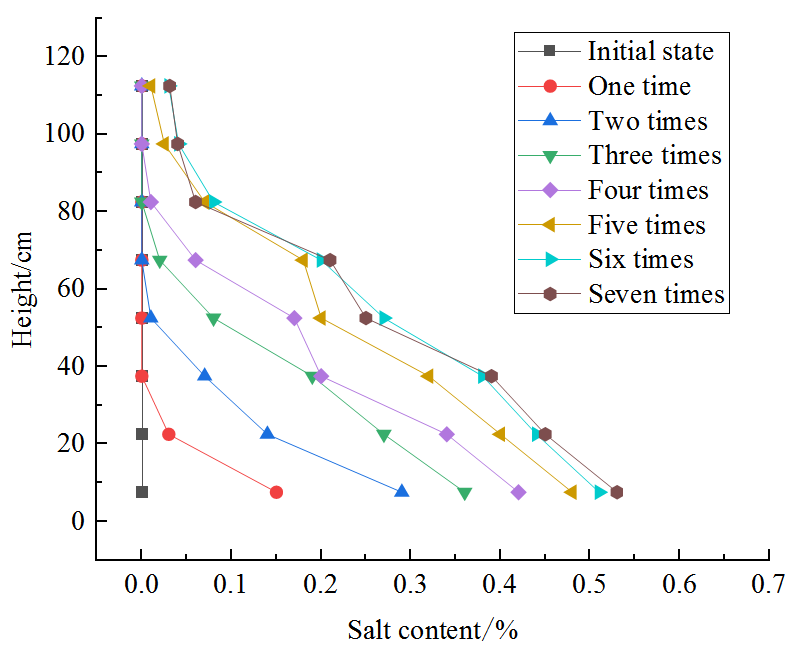


| Height | Initial state | One time | Two times | Three  times | Four  times | Five  times | Six  times | Seven  times |
| --- | --- | --- | --- | --- | --- | --- | --- | --- |
| 112.5 | 0 | 0 | 0 | 0 | 0 | 0.01 | 0.03 | 0.031 |
| 97.5 | 0 | 0 | 0 | 0 | 0 | 0.025 | 0.041 | 0.04 |
| 82.5 | 0 | 0 | 0 | 0 | 0.01 | 0.07 | 0.08 | 0.06 |
| 67.5 | 0 | 0 | 0 | 0.02 | 0.06 | 0.18 | 0.2 | 0.21 |
| 52.5 | 0 | 0 | 0.01 | 0.08 | 0.17 | 0.2 | 0.27 | 0.25 |
| 37.5 | 0 | 0 | 0.07 | 0.19 | 0.2 | 0.32 | 0.38 | 0.39 |
| 22.5 | 0 | 0.03 | 0.14 | 0.27 | 0.34 | 0.4 | 0.44 | 0.45 |
| 7.5 | 0 | 0.15 | 0.29 | 0.36 | 0.42 | 0.48 | 0.51 | 0.53 |

Figure 11:

1.
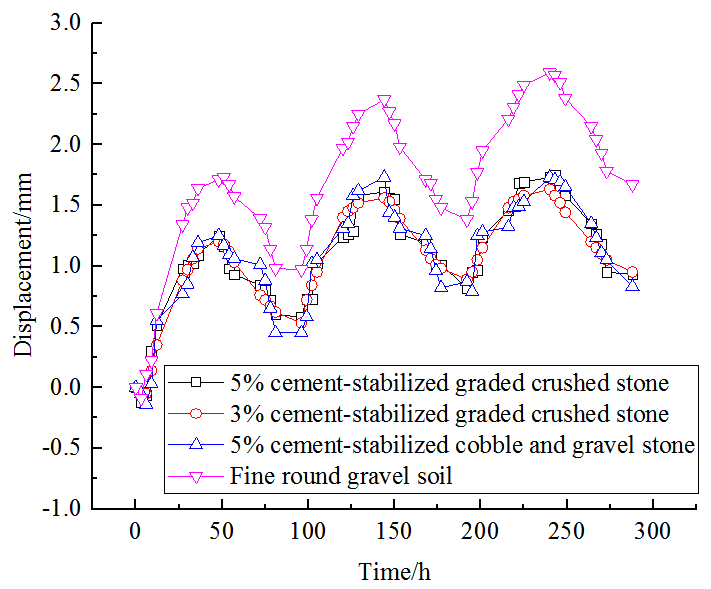


| Time | Displacement |  |  |  |
| --- | --- | --- | --- | --- |
| h | mm |  |  |  |
|  | 5% cement-stabilized graded crushed stone | 3% cement-stabilized graded crushed stone | 5% cement-stabilized cobble and gravel stone | Fine round gravel stone |
| 0 | 0 | 0 | 0 | 0 |
| 3 | -0.12 | -0.04 | -0.05 | -0.09 |
| 6 | -0.06 | -0.05 | -0.14 | 0.11 |
| 9 | 0.3 | 0.14 | 0.03 | 0.22 |
| 12 | 0.51 | 0.35 | 0.55 | 0.61 |
| 27 | 0.98 | 0.88 | 0.77 | 1.34 |
| 30 | 1.01 | 0.97 | 0.85 | 1.48 |
| 33 | 1.02 | 1.06 | 1.07 | 1.52 |
| 36 | 1.09 | 1.14 | 1.19 | 1.64 |
| 48 | 1.25 | 1.2 | 1.25 | 1.71 |
| 51 | 1.16 | 1.18 | 1.17 | 1.73 |
| 54 | 0.98 | 1.12 | 1.09 | 1.67 |
| 57 | 0.93 | 1.03 | 1.06 | 1.57 |
| 72 | 0.84 | 0.76 | 1.01 | 1.39 |
| 75 | 0.8 | 0.72 | 0.88 | 1.32 |
| 78 | 0.72 | 0.67 | 0.65 | 1.14 |
| 81 | 0.6 | 0.62 | 0.45 | 0.98 |
| 96 | 0.58 | 0.53 | 0.45 | 0.97 |
| 99 | 0.73 | 0.72 | 0.58 | 1.14 |
| 102 | 0.73 | 0.84 | 1.02 | 1.38 |
| 105 | 1.02 | 0.95 | 1.05 | 1.56 |
| 120 | 1.24 | 1.4 | 1.31 | 1.97 |
| 123 | 1.26 | 1.45 | 1.35 | 2.02 |
| 126 | 1.29 | 1.48 | 1.58 | 2.15 |
| 129 | 1.58 | 1.52 | 1.62 | 2.25 |
| 144 | 1.61 | 1.56 | 1.73 | 2.37 |
| 147 | 1.56 | 1.53 | 1.44 | 2.27 |
| 150 | 1.55 | 1.46 | 1.4 | 2.17 |
| 153 | 1.26 | 1.39 | 1.31 | 1.98 |
| 168 | 1.19 | 1.13 | 1.25 | 1.71 |
| 171 | 1.08 | 1.06 | 1.14 | 1.68 |
| 174 | 1.01 | 1.01 | 0.96 | 1.55 |
| 177 | 1.01 | 0.98 | 0.82 | 1.48 |
| 192 | 0.82 | 0.89 | 0.87 | 1.38 |
| 195 | 0.95 | 0.95 | 0.79 | 1.53 |
| 198 | 0.97 | 1.05 | 1.25 | 1.77 |
| 201 | 1.24 | 1.15 | 1.28 | 1.95 |
| 216 | 1.46 | 1.48 | 1.32 | 2.21 |
| 219 | 1.5 | 1.53 | 1.48 | 2.31 |
| 222 | 1.68 | 1.55 | 1.49 | 2.41 |
| 225 | 1.69 | 1.58 | 1.53 | 2.49 |
| 240 | 1.73 | 1.63 | 1.73 | 2.59 |
| 243 | 1.75 | 1.58 | 1.71 | 2.57 |
| 246 | 1.65 | 1.52 | 1.69 | 2.51 |
| 249 | 1.58 | 1.44 | 1.65 | 2.38 |
| 264 | 1.35 | 1.2 | 1.35 | 2.15 |
| 267 | 1.26 | 1.15 | 1.23 | 2.04 |
| 270 | 1.18 | 1.1 | 1.11 | 1.93 |
| 273 | 0.95 | 1.05 | 1.05 | 1.78 |
| 288 | 0.93 | 0.95 | 0.83 | 1.67 |

1.
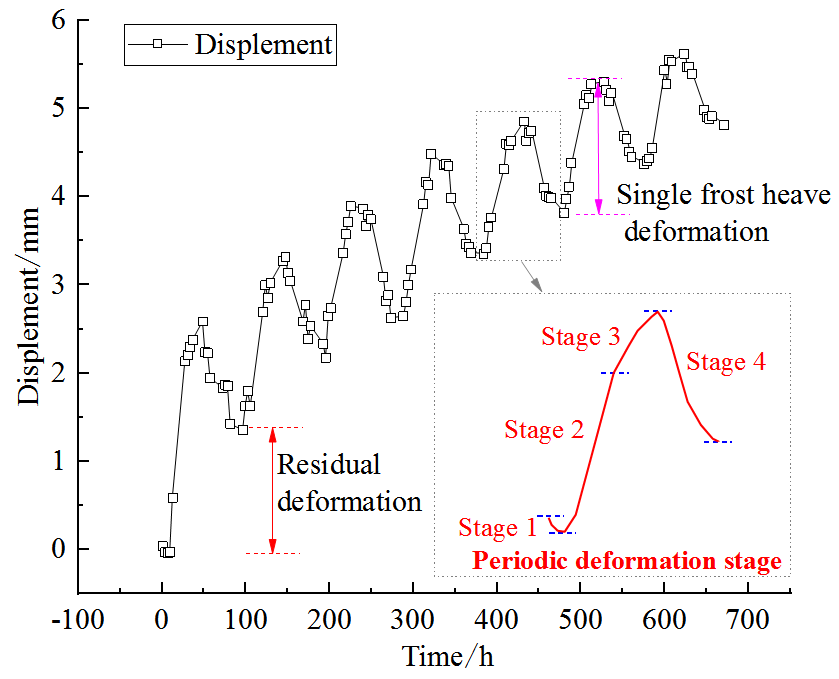


| Time | Displacement |
| --- | --- |
| h | mm |
| 0 | 0.04 |
| 3 | -0.03 |
| 6 | -0.04 |
| 9 | -0.03 |
| 12 | 0.59 |
| 27 | 2.14 |
| 30 | 2.21 |
| 33 | 2.3 |
| 36 | 2.38 |
| 48 | 2.59 |
| 51 | 2.24 |
| 54 | 2.23 |
| 57 | 1.95 |
| 72 | 1.84 |
| 75 | 1.87 |
| 78 | 1.86 |
| 81 | 1.43 |
| 96 | 1.36 |
| 99 | 1.63 |
| 102 | 1.8 |
| 105 | 1.63 |
| 120 | 2.7 |
| 123 | 3 |
| 126 | 2.86 |
| 129 | 3.03 |
| 144 | 3.28 |
| 147 | 3.32 |
| 150 | 3.14 |
| 153 | 3.05 |
| 168 | 2.59 |
| 171 | 2.78 |
| 174 | 2.39 |
| 177 | 2.54 |
| 192 | 2.34 |
| 195 | 2.18 |
| 198 | 2.65 |
| 201 | 2.74 |
| 216 | 3.37 |
| 219 | 3.58 |
| 222 | 3.72 |
| 225 | 3.9 |
| 240 | 3.87 |
| 243 | 3.67 |
| 246 | 3.8 |
| 249 | 3.75 |
| 264 | 3.1 |
| 267 | 2.82 |
| 270 | 2.89 |
| 273 | 2.63 |
| 288 | 2.65 |
| 291 | 2.81 |
| 294 | 3.01 |
| 297 | 3.18 |
| 312 | 3.92 |
| 315 | 4.17 |
| 318 | 4.14 |
| 321 | 4.49 |
| 336 | 4.37 |
| 339 | 4.38 |
| 342 | 4.35 |
| 345 | 3.99 |
| 360 | 3.64 |
| 363 | 3.47 |
| 366 | 3.44 |
| 369 | 3.37 |
| 384 | 3.36 |
| 387 | 3.42 |
| 390 | 3.66 |
| 393 | 3.77 |
| 408 | 4.32 |
| 411 | 4.61 |
| 414 | 4.59 |
| 417 | 4.64 |
| 432 | 4.86 |
| 435 | 4.64 |
| 438 | 4.73 |
| 441 | 4.75 |
| 456 | 4.11 |
| 459 | 4.02 |
| 462 | 4 |
| 465 | 3.99 |
| 480 | 3.82 |
| 483 | 3.98 |
| 486 | 4.12 |
| 489 | 4.39 |
| 504 | 5.06 |
| 507 | 5.16 |
| 510 | 5.13 |
| 513 | 5.28 |
| 528 | 5.31 |
| 531 | 5.22 |
| 534 | 5.09 |
| 537 | 5.18 |
| 552 | 4.7 |
| 555 | 4.66 |
| 558 | 4.52 |
| 561 | 4.46 |
| 576 | 4.38 |
| 579 | 4.41 |
| 582 | 4.44 |
| 585 | 4.56 |
| 600 | 5.44 |
| 603 | 5.29 |
| 606 | 5.56 |
| 609 | 5.54 |
| 624 | 5.63 |
| 627 | 5.48 |
| 630 | 5.48 |
| 633 | 5.4 |
| 648 | 4.99 |
| 651 | 4.91 |
| 654 |  |
| 657 | 4.92 |
| 672 | 4.82 |

Figure 12:


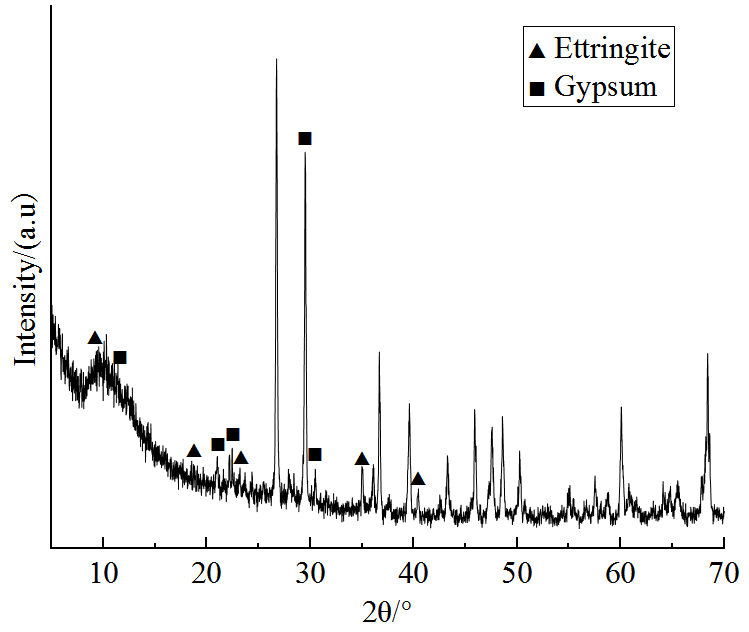

Supplement: S1 Data — (DOCX) [file pone.0337173.s001.docx]
