# Supplementary material for: Experimental study on water-heat-salt migration and deformation characteristics of subgrade filler during freeze-thaw cycles in Northwestern China
Source: PLoS One. 2025 Dec 4;20(12):e0337173. doi: 10.1371/journal.pone.0337173 (PMC12677788; doi:10.1371/journal.pone.0337173)
Supplement: Attachment — Submitted filename: Response to Reviewers.docx [file pone.0337173.s003.docx]

Response to Reviewers

Dear editors,

Thank you for your valuable feedback and constructive suggestions on our manuscript, [Experimental study on water-heat-salt migration and deformation characteristics of subgrade filler during freeze-thaw cycles]. We sincerely appreciate the time and effort you and the reviewers have dedicated to evaluating our work.

In response to your comments, we have carefully revised the manuscript to address the points raised. Specifically, we have:

Thoroughly reorganized the content for improved clarity and flow.

Reformatted the manuscript to comply fully with the journal’s guidelines.

We believe these revisions have significantly enhanced the quality of the paper, and we are grateful for your insightful guidance. Please find our revised manuscript attached for your consideration. Should there be any further adjustments required, we would be happy to address them promptly.

Thank you once again for your support. We look forward to your feedback.

Best regards,

**Reviewer #1:** Experimental study on water-heat-salt migration and deformation characteristics of subgrade filler during freeze-thaw cycles
**Abstract**
• The abstract provides a broad summary of the study, covering the motivation, methodology, and findings. However, the structure is imbalanced. The rationale (arching in culverts due to freeze-thaw cycles) is introduced well, but the objective is not clearly stated as a standalone sentence.

It has been corrected. The objective is stated: ”The seasonal freeze-thaw action of saline soils in Northwestern China deteriorates embankment material properties, thereby adversely affecting train ride comfort and increasing railway maintenance expenditures. ”

• The methods are implied but not concisely defined (e.g., freeze-thaw tests on soil columns).

Freeze-Thaw Tests on Soil Columns refer to laboratory experiments designed to simulate and evaluate the effects of cyclic freezing and thawing on the engineering properties of soil specimens configured in geometry. These tests are critical in moisture migration, and structural stability in northwestern regions.

• The findings are detailed, but too verbose for an abstract. Quantitative data is partially presented but would benefit from clearer summaries (e.g., numerical deformation values, migration rates).

It has been corrected.

• Clearly identify the research objective and condense findings into 2–3 data-backed sentences.

It has been corrected.

The results show that the magnitudes of temperature variations decrease upon increasing the distance from the top. Additionally, under alternating hot and cold cycles, a temperature lag occures at the lower sensors. For the multi-layer soil column, the water content increases with the number of freeze-thaw cycles. The salt content in the multi-layer soil column gradually migrates upward upon increasing the number of freeze-thaw cycles, and the overall salt content continuously increases, reaching the top of the column by the 5th cycle. During both single-layer and multi-layer freeze-thaw cycles, the accumulated residual deformation increases with the number of cycles. Component analysis and microstructural examinations reveal the formation of ettringite and gypsum within the samples, which are related to water-heat-salt migration.

• Clearly articulate the objective of the study as a standalone sentence.

It has been corrected. The objective of the study is stated as : “The seasonal freeze-thaw action of saline soils in Northwestern China deteriorates embankment material properties, thereby adversely affecting train ride comfort and increasing railway maintenance expenditures.”

• Condense the description of findings; include only the most critical quantitative results.

It has been corrected.

• Maintain balance by briefly mentioning the methodology and limiting excessive detail in the findings.

It has been corrected.

**Introduction**
• The hypothesis and aim are implied but should be explicitly stated at the end of the section.

Thank you for your kind suggestions, the aims have be explicitly stated at the end of the introduction.
• Streamline the literature review by focusing on studies most relevant to multilayer subgrade behavior.

The literatures have be streamlined.
• Show the quantitative data of previous works and how they differ from your study.

The study focus on the frost heave disease occuring at transition section of a ballastless railway embankment in northwest China, which is missing from previous literature. Therefore, the vertical migration patterns of water- heat-salt in subgrade fillers are analyzed through laboratory freeze-thaw cycles. The arching mechanism in saline soil on the subgrade transition section areas is revealed. The results are expected to provide engineering references for similar projects in the region.
• This study suggested to be used:
Saleh, S. A., Ismael, R. S. ., & Abas, B. S. . (2024). Effect of Soil Stabilization on Structural Design of Flexible Pavement. Journal of Studies in Civil Engineering, 1(1), 36–54. https://doi.org/10.53898/jsce2024113

Thank you for your suggestions, the reference format have be corrected.
**Experimental Materials and Scheme**
• Include a summary table detailing the freeze-thaw cycle parameters (temperature range, duration, number of cycles).

The upper part of the sample is subjected to a temperature cycle of -30 oC to 40 oC, with the soil column model placed in a temperature-controlled room at 20 oC, while the lower part is maintained at room temperature. Each cycle consists of 96 hours, with 48 hours of freezing (-30 oC) followed by 48 hours of thawing (40 oC). The single-layer and multi-layer soil column freeze-thaw cycling tests are conducted for 3 and 7 freeze-thaw cycles, respectively.
• Clarify the rationale for choosing specific cement stabilization percentages across materials.

For the 3% cement-stabilized graded crushed stone, 5% cement-stabilized cobble and gravel stone, and fine round gravel soil., those materials are selected in accordance with the railway subgrade design specifications.

• Mention whether replicate tests were conducted to ensure reproducibility.

The replicate tests are conducted to ensure the reproducibility.
**Experimental Results and Analysis**
**Temperature Variation**
• Support observed trends (e.g., temperature lag) with statistical validation or replicating data.

• Highlight how the findings align or contrast with existing models of thermal migration in soils.

Current research on water-heat-salt migration primarily focuses on the freeze-thaw cycle-induced water-heat-salt migration and salt expansion characteristics in fine-grained soils, with limited studies addressing the impact of sulfate erosion on hydrothermal salt migration in subgrade soils. The distinctiveness of this study lies in conducting laboratory-scale model experiments to investigate the vertical migration patterns of hydrothermal salts in subgrade fillers under freeze-thaw cycles
**Moisture Content**
• Provide a comparative table summarizing moisture change across materials and cycle numbers.

The relevant data are presented in Fig 4 and Fig 5.

Fig 4 shows the variations in moisture content with height after three freeze-thaw cycles for different fillers.

The moisture content variation with height after different freeze-thaw cycles for the multi-layer soil column is shown in Fig 5.

• Consider discussing the implications of water redistribution on structural integrity more directly.

It has corrected.
**Salt Content**
• Expand discussion on the practical impact of upward salt migration, especially in upper structural layers.

It has corrected.

After the first freeze-thaw cycle, there is a significant change in salt content in the lower part and a minor change in the upper part due to the rapid freezing process where salt does not have enough time to migrate upwards. During the cold cycle, water above the freezing front formes ice crystals, causing salt to accumulate near the front and preventing diffusion into solid ice, resulting in a minor change in salt content in the upper part. The lower part has a supply of salt solution, leading to a greater change in the salt content due to the gradient effect and the continuous migration of water and salt. As the number of freeze-thaw cycles increases, the change in salt content decreases in the lower part, with significant changes mainly occurring in the middle of the soil column. This is because the continuous migration of water and salt intensifies, forming migration channels within the soil, and the melting of ice crystals in the upper part during the thaw cycle increases the moisture content and amount of dissolved salt. Some salt dissolves in water due to gravity and migrates downwards, resulting in a lower salt content in the upper part.
• Include statistical ranges or error bars in salt content measurements for clarity.

Before the test, the components were calibrated. The relevant error analysis is not involved here.

**Deformation Analysis**
• Present residual deformation results in tabular form for quick comparison across materials.

It has corrected.
•
Discuss how the four-stage deformation process informs design recommendations for embankments in similar regions.

It has increased.

In engineering sites, the temperature difference can be controlled to reduce the deformation at each stage.

**Microanalysis**
• Quantify microstructural features where possible (e.g., pore sizes, crystal dimensions).

The content and size of pore channels are random, and the relevant parameters have not been analyzed here.

• Consider correlating SEM/XRD findings more directly to macro-scale deformation behaviors.

During the cold cycle, salt precipitates, and upon increasing the temperature, the salt redissolves. During continuous freeze-thaw cycles, the salt and precipitates undergoes repeated precipitation-dissolution, which changes the soil structure. Soil particles experiences relative displacement, and pores gradually form during water-salt migration, which serve as continuous migration channels.

• Specify the representativeness of the samples analyzed (e.g., selection criteria).

The content and size of pore channels are random, and the analysis of relevant components is universal.

**Conclusion**
• Condense the list of findings and focus on the most impactful results.

It has corrected.

In single-layer soil columns with different fillers, the moisture content decreases upon increasing the height. For multi-layer soil column, the moisture content generally increases with the freeze-thaw cycles, but the rate of increase slows as the number of cycles increase. The moisture content curve shows higher values at both ends and lower values in the middle. During warm cycles, waterlogging occurs inside the soil column, while freezing fronts and cap effects occurs during cold cycles.

In single-layer soil columns, the salt content decreases upon increasing the height for different fillers. For multi-layer soil column, salt migrates upwards during the freeze-thaw cycles, and the overall salt content increases continuously. During cold cycles, the freezing front also hinders salt migration to some extent. In both single-layer and multi-layer freeze-thaw cycles, the cumulative residual deformation increases with the number of cycles, but the rate of increase gradually slows. The freeze-thaw process mainly consists of a cooling and shrinkage stage, a salt expansion-erosion stage, a salt expansion-erosion-freezing expansion stage, and a warming and melting stage.

XRD analysis reveals the simultaneous generation of ettringite and gypsum within the samples. SEM analysis shows that the soil particles are covered with a layer of cementitious material that include some precipitated columnar ettringite and gypsum crystals due to water-temperature-salt migration.
• Add a final paragraph summarizing engineering implications and potential field applications.

It has increased.

In the soil column freeze-thaw cycling tests , only a single type of cement was used. Although the cement produced by different regions and manufacturers had the same grade, there were still some differences in their chemical compositions. The chemical reaction of sulfate erosion on cement-mixed graded crushed stones can still be analyzed qualitatively and quantitatively in detail.

• Briefly mention any limitations of the study and suggest future research directions, such as long-term field validation or model development.

It has increased.

On this basis, various prevention and control measures can be developed to prevent related diseases. It is worth further research to start from multiple aspects such as physics and chemistry.

**Reviewer #2:**

**1.Title and Abstract**
The title is informative but could be more specific. For example, mention the geographic context (e.g., "in Northwestern China") or emphasize the sulfate erosion aspect more clearly. The abstract is dense and includes too many technical details without sufficient explanation of their significance. Can the abstract be revised to clearly define the research problem, methods, key findings, and implications in a more reader-friendly format? What is meant by “arching disease” in the abstract? Consider defining this for readers unfamiliar with the term.

It has corrected, the objective is emphasized more clearly: ”The seasonal freeze-thaw action of saline soils in Northwestern China deteriorates embankment material properties, thereby adversely affecting train ride comfort and increasing railway maintenance expenditures. ” The abstract is revised to clearly define the research problem, methods, key findings and implications. The meaning of “arching disease” is the phenomenon of upward heaving deformation in the subgrade. In the seasonal saline soil regions of northwestern China, the presence of sulfate saline soils at the surface can severely impact the service performance of subgrade fill materials under the combined effects of freeze-thaw cycles and sulfate erosion, manifesting as upward heaving deformation of the subgrade.

**2. Introduction**
The introduction provides a solid literature foundation but lacks a concise statement of the knowledge gap and hypothesis.

The study focus on the frost heave disease occuring at transition section of a ballastless railway embankment in northwest China, which is missing from previous literature. Therefore, the vertical migration patterns of water- heat-salt in subgrade fillers are analyzed through laboratory freeze-thaw cycles. The arching mechanism in saline soil on the subgrade transition section areas is revealed. The results are expected to provide engineering references for similar projects in the region.
The "arching disease" is mentioned but not well contextualized.

The meaning of “arching disease” is the phenomenon of upward heaving deformation in the subgrade. In the seasonal saline soil regions of northwestern China, the presence of sulfate saline soils at the surface can severely impact the service performance of subgrade fill materials under the combined effects of freeze-thaw cycles and sulfate erosion, manifesting as upward heaving deformation of the subgrade.

Can the authors clearly articulate the specific research questions or hypotheses that this study seeks to answer?

In the setting of the frost heave disease occuring at transition section of a ballastless railway embankment in northwest China. The vertical migration patterns of water- heat-salt in subgrade fillers are analyzed through laboratory freeze-thaw cycles. The arching mechanism in saline soil on the subgrade transition section areas is revealed. The results are expected to provide engineering references for similar projects in the region.
What distinguishes this study from the numerous prior studies cited on water-salt migration and sulfate erosion? Can the mechanism or definition of “arching disease” in transition sections be illustrated with a figure or schematic?

Current research on water-heat-salt migration primarily focuses on the freeze-thaw cycle-induced water-heat-salt migration and salt expansion characteristics in fine-grained soils, with limited studies addressing the impact of sulfate erosion on hydrothermal salt migration in subgrade soils. The distinctiveness of this study lies in conducting laboratory-scale model experiments to investigate the vertical migration patterns of hydrothermal salts in subgrade fillers under freeze-thaw cycles. In the seasonal saline soil regions of northwestern China, the presence of sulfate saline soils at the surface can severely impact the service performance of subgrade fill materials under the combined effects of freeze-thaw cycles and sulfate erosion, manifesting as upward heaving deformation of the subgrade.

**3.Experimental Materials and Scheme**
The rationale for selecting the specific filler types and stabilization percentages is not explained. Moisture contents appear unusually low (e.g., 3–5%). Clarify if this refers to dry densities or actual field conditions.

The moisture content is controlled around 4.75%, 5.51%, 4.38%, and 3.07%. Layered compaction is carried out using a homemade circular tamper to achieve a compaction degree similar to those in the field.

What criteria guided the selection of 3% and 5% cement contents? Are these field-validated values?

This represents the standard dosage of conventional subgrade fill materials.
Why was 7 cycles chosen for the multi-layer test and 3 for the single-layer? Is there a theoretical or practical justification?

During the test process, for single-layer tests, based on the fact that water and salt from the lower part can migrate to the top, the number of freeze-thaw cycles is relatively small. For multi-layer tests, based on the migration of water and salt from the lower layer to the first upper layer, the number of freeze-thaw cycles is relatively large. The focus is on analyzing the migration patterns of moisture and salt with the number of freeze-thaw cycles.

Is the 96-hour cycle duration realistic compared to field freeze-thaw rates?

From a practical engineering perspective, the 96-hour cycle is deemed feasible.
**4. Results and Analysis**
Temperature Variation
Figures 2 and 3 are helpful but need better labeling and scale uniformity (legends? The numbering should be defined).

It has corrected.
The description of "temperature lag" lacks quantification.Can the authors quantify the temperature lag across sensors and compare it with model predictions or literature values? Were thermal properties of the materials (e.g., thermal conductivity) considered in interpreting the lag?

For this test, the temperature lags behind the relevant phenomena obtained under specific test conditions (such as temperature changes, heat conduction parameters, etc.). The heat conduction parameters of each material were not determined, and no quantitative research was conducted.

**5. Moisture Content Variation**
The “high-low-high” water distribution is interesting but not fully explained.

It has corrected.
The concept of "water lag" and "lid effect" is not defined clearly.

After freeze-thaw cycles were applied to the soil column, the temperature-insensitive soil at the lower section exhibited minimal thermal influence. Following the infiltration of externally supplied solution into the soil matrix, its migration tendency significantly attenuated. Concurrently, moisture within the soil evaporated into the atmosphere. As the freeze-thaw system entered the cooling phase, the temperature at the column's top decreased, causing atmospheric moisture to condense on the soil surface. This process resulted in “water lag” and the “lid effect”.
What mechanisms contribute to the “high-low-high” pattern: capillarity, thermal gradient, or osmotic flow?

During the cooling phase of , this induces upward moisture migration towards the colder upper region. , moisture consistently migrates from the warm end to the cold end. Specifically during cooling cycles, water from lower sections migrates upwards. After 48 hours of upward migration during the cooling phase, when the system transitions to the heating phase, the temperature in the upper soil column rises. During the cooling phase of cyclic freezing-thawing, moisture migrates towards the upper region. Throughout the freeze-thaw cycling process, moisture migrates from the warm end to the cold end, and during the cold cycles, moisture from the bottom migrates upwards. After 48 hours of upward migration, the moisture enters the hot cycle as the temperature at the top of the soil column increases. This causes thawing of previously frozen soil, where ice crystals between soil particles melt into liquid water and subsequently percolate downward due to gravitational forces.
How does the presence of salts affect freezing/melting behavior and water redistribution?

During freeze-thaw cycling, the melted ice crystals within the soil matrix transform into liquid water that percolates downward through the frozen soil zone under gravitational forces. Concurrently, the formation of ice crystals in the frozen soil, coupled with precipitated salt crystals resulting from temperature-induced solute exclusion, progressively occlude the interparticle voids. This dual process ultimately leads to the obstruction of water and solute migration pathways, which affect freezing/melting behavior and water redistribution.
**6. Salt Content Variation**
Salt migration is well documented but would benefit from mass balance or modeling discussion. The observed salt redistribution appears counterintuitive in later stages. Can the authors provide a salt mass balance over cycles to show conservation or net accumulation?

This indoor test was designed in the same way as the field test, with an unlimited supply of salt water at the bottom, resulting in an overall increase in salt content throughout the test.

How do salt precipitation/dissolution dynamics (e.g., solubility curves) explain the observed redistribution?

The salt content was calibrated for relevant components before the test. Changes in salt content can cause variations in the conductivity coefficient.

**7. Deformation Analysis**
The division into four freeze-thaw stages is insightful. Deformation behavior is mainly described qualitatively.
Can the axial displacement be correlated with water/salt content or microstructural changes? How repeatable or statistically significant are these deformation measurements?

The replicate tests are conducted to ensure the reproducibility.
**8. Microanalysis**
XRD and SEM results are interesting but poorly integrated with mechanical behavior analysis. No discussion of quantitative phase analysis or crystal orientation/density. What is the estimated amount of ettringite and gypsum formed, and how does this relate to observed swelling? Is there any SEM or EDS evidence showing salt crystallization blocking pore throats?

XRD and SEM results can qualitatively analyze the causes of deformation in terms of composition and microstructure. The XRD shows the reaction between calcium ions in the cement hydration products and sulfate ions in the solution forms gypsum, which subsequently reacts with calcium hydroxide in the cement hydration products to form ettringite. When the concentration of sulfate ions in the solution is high, the chemical reaction to produce gypsum continues, resulting in residual gypsum during the formation of ettringite, which precipitates simultaneously with ettringite. SEM results show the soil particles are tightly connected, with a layer of cementitious material covering their surface, filling the gaps between soil particles. Additionally, there are pores of varying sizes between soil particles. During the cold cycle, salt precipitates, and upon increasing the temperature, the salt redissolves. During continuous freeze-thaw cycles, the salt and precipitates undergoes repeated precipitation-dissolution, which changes the soil structure. Soil particles experiences relative displacement, and pores gradually form during water-salt migration, which serve as continuous migration channels. Upon further magnification, short columnar ettringite and gypsum crystals precipitate, which disrupt the connectivity between soil particles, leading to the formation of larger pores. Flocculent precipitates appeare between crystal particles, with a significant amount of salt precipitates adhering to the surface of soil particles and filling some of the pores. This blocks some of the water-salt migration channels, thereby inhibiting water-salt migration.

**9. Discussion (Missing/Underdeveloped)**
There is no dedicated discussion section to synthesize findings or relate them to broader engineering practice.
What are the practical implications of these findings for high-speed railway subgrade design in saline soil regions?
How can the insights on moisture/salt migration inform predictive modeling or mitigation strategies?

In the saline soil regions of northwestern China, freeze-thaw cycling induces subgrade heaving deformation, which subsequently compromises operational safety of rail infrastructure. In the study, The vertical migration patterns of water-heat-salt in subgrade fillers are analyzed through laboratory freeze-thaw cycles. The arching mechanism in saline soil on the subgrade transition section areas is revealed. The results are expected to provide engineering references for similar projects in the region
**10. Conclusion**
The conclusions are mostly restatements of results. Lacks discussion on limitations and future work. Can the authors highlight the main engineering recommendations derived from this study? What are the main limitations (e.g., lab scale vs. field scale, duration, boundary conditions)?

It has corrected.
**11. References and Style**
The citation style is inconsistent (e.g., use of Chinese references with limited access). Some citations appear repetitive or too many from the same group. Can the authors diversify references with more international studies or review papers?

Thank you for your kind suggestions, the references have checked and the citation style is consistent now.
**12. General/Editorial Suggestions**
Improve language clarity: Terms like "occurres" and “slowes” suggest grammar and spell-checking is needed. Add schematics of the experimental setup and sensor arrangement. Ensure all figures are legible with consistent scales and labels.

Thank you for your kind suggestions, the grammar and spell-checking is conducted, and others are corrected.
