# Supplementary material for: Experimental study on water-heat-salt migration and deformation characteristics of subgrade filler during freeze-thaw cycles in Northwestern China
Source: PLoS One. 2025 Dec 4;20(12):e0337173. doi: 10.1371/journal.pone.0337173 (PMC12677788; doi:10.1371/journal.pone.0337173)
Supplement: Attachment — Submitted filename: Response to the Review Comments.docx [file pone.0337173.s004.docx]

**Comments to the Author**

1. If the authors have adequately addressed your comments raised in a previous round of review and you feel that this manuscript is now acceptable for publication, you may indicate that here to bypass the “Comments to the Author” section, enter your conflict of interest statement in the “Confidential to Editor” section, and submit your "Accept" recommendation.

Reviewer #1: All comments have been addressed

2. Is the manuscript technically sound, and do the data support the conclusions?

The manuscript must describe a technically sound piece of scientific research with data that supports the conclusions. Experiments must have been conducted rigorously, with appropriate controls, replication, and sample sizes. The conclusions must be drawn appropriately based on the data presented.

Reviewer #1: Yes

3. Has the statistical analysis been performed appropriately and rigorously?

Reviewer #1: N/A

4. Have the authors made all data underlying the findings in their manuscript fully available?

The PLOS Data policy requires authors to make all data underlying the findings described in their manuscript fully available without restriction, with rare exception (please refer to the Data Availability Statement in the manuscript PDF file). The data should be provided as part of the manuscript or its supporting information, or deposited to a public repository. For example, in addition to summary statistics, the data points behind means, medians and variance measures should be available. If there are restrictions on publicly sharing data—e.g. participant privacy or use of data from a third party—those must be specified.

Reviewer #1: Yes

5. Is the manuscript presented in an intelligible fashion and written in standard English?

PLOS ONE does not copyedit accepted manuscripts, so the language in submitted articles must be clear, correct, and unambiguous. Any typographical or grammatical errors should be corrected at revision, so please note any specific errors here.

Reviewer #1: Yes

**Review Comments to the Author**

Please use the space provided to explain your answers to the questions above. You may also include additional comments for the author, including concerns about dual publication, research ethics, or publication ethics. (Please upload your review as an attachment if it exceeds 20,000 characters)

Reviewer #1: Thank you for revising the manuscript and for carefully responding to the reviewers’ comments. The revision is appreciated. However, I would like to suggest two points for further improvement:

1. In the response to each comment, it would be better to indicate the page and line numbers where the modifications have been made in the revised version. This will help the reviewers easily identify the changes and avoid any confusion.

Two references are added on page 13, which I have indicated so that the reviewers can easily identify the changes and avoid confusion.

2. The number of references should be increased where appropriate. Strengthening the literature support will improve the overall quality of the manuscript. I recommend adding the following references in the revised version:

Saleh, S. A., Ismael, R. S., & Abas, B. S. (2024). Effect of Soil Stabilization on Structural Design of Flexible Pavement. Journal of Studies in Civil Engineering, 1(1), 36–54. https://doi.org/10.53898/jsce2024113

Shirmohammadi, M., Hakimi Asiabar, S., and Peyrovi Cheshnasar, M. (2025). Dual Transitional Floors and Soil-Structure Synergy: A Paradigm Shift in Seismic Resilience for Hybrid High-Rise Systems. Advances in Civil Engineering and Environmental Science, 2(2), 62-71. doi: 10.22034/acees.2025.508498.1021

Thank you for your kind suggestions, the references have added in the revised version to improve the overall quality of the manuscript.

7. PLOS authors have the option to publish the peer review history of their article (what does this mean?). If published, this will include your full peer review and any attached files.

If you choose “no”, your identity will remain anonymous but your review may still be made public.

Do you want your identity to be public for this peer review? For information about this choice, including consent withdrawal, please see our Privacy Policy.

Reviewer #1: No
